# *Streptomyces* spp. From the Marine Sponge *Antho dichotoma*: Analyses of Secondary Metabolite Biosynthesis Gene Clusters and Some of Their Products

**DOI:** 10.3389/fmicb.2020.00437

**Published:** 2020-03-18

**Authors:** Jaime Felipe Guerrero-Garzón, Martin Zehl, Olha Schneider, Christian Rückert, Tobias Busche, Jörn Kalinowski, Harald Bredholt, Sergey B. Zotchev

**Affiliations:** ^1^Department of Pharmacognosy, University of Vienna, Vienna, Austria; ^2^Department of Analytical Chemistry, Faculty of Chemistry, University of Vienna, Vienna, Austria; ^3^Center for Biotechnology, Bielefeld University, Bielefeld, Germany; ^4^Xellia Pharmaceuticals AS, Oslo, Norway

**Keywords:** marine sponge, *Streptomyces* spp., genomics, biosynthetic gene clusters, horizontal gene transfer, secondary metabolites

## Abstract

Actinomycete bacteria from marine environments represent a potential source for new antibiotics and anti-tumor drugs. Ten strains belonging to the genus *Streptomyces* isolated from the marine sponge *Antho dichotoma* collected at the bottom of the Trondheim fjord (Norway) were screened for antibiotic activity. Since only few isolates proved to be bioactive in the conditions tested, we decided to gain an insight into their biosynthetic potential using genome sequencing and analysis. Draft genomes were analyzed for the presence of secondary metabolite biosynthesis gene clusters (BGCs) using antiSMASH software. BGCs specifying both known and potentially novel secondary metabolites were identified, suggesting that these isolates might be sources for new bioactive compounds. The results of this analysis also implied horizontal transfer of several gene clusters between the studied isolates, which was especially evident for the lantibiotic- and thiopeptide-encoding BGCs. The latter implies the significance of particular secondary metabolites for the adaptation of *Streptomyces* to the spatially enclosed marine environments such as marine sponges. Two bioactive isolates, one showing activity against both yeast and *Bacillus subtilis*, and one only against yeast were analyzed in details, leading to the identification of cycloheximide, linearmycins, and echinomycins that are presumably responsible for the observed bioactivities.

## Introduction

*Streptomyces* are Gram-positive GC rich bacteria of the order Actinomycetales that are ubiquitous in nature and can be isolated from a variety of sources, including terrestrial (soil, insects, animals and plants), as well as marine (sediments, fish, corals and sponges) habitats (Goodfellow et al., 2018; Subramani and Sipkema, 2019). These bacteria remain of significant interest in terms of discovery of biologically active secondary metabolites that can potentially be developed into human medicines such as antimicrobial and anticancer agents, immunosuppressants etc. (Takahashi and Nakashima, 2018). The unprecedented genetic potential of *Streptomyces* to produce diverse secondary metabolites is exemplified by a large number (usually 20–40) of biosynthetic gene clusters harbored by their genomes (Baltz, 2017). These clusters are usually tightly controlled by sophisticated regulatory networks, and in the laboratory conditions, only a few of them are expressed at the level allowing purification and analyses of the product (Reen et al., 2015). It is tempting to speculate that specific environmental signals are required to induce expression of particular clusters, thus leading to the production of secondary metabolites that would give a certain competitive advantage to the producing organisms. Recent comparative genomics-based studies of *Streptomyces* and other actinomycete bacteria revealed that representatives of the same species isolated from different environments have different sets of secondary metabolite biosynthesis gene clusters (BGCs) (Ian et al., 2014; Ziemert et al., 2014). Consequently, it is plausible that when a particular strain is transferred to a new environment (e.g., by birds, animals, rivers, and ocean currents etc.), some BGCs are lost, while others, specifying production of compounds beneficial for adaptation, are being acquired. Although the exact method of acquisition can vary (e.g., natural transformation, conjugative transfer or transduction), it seems likely that other actinomycete bacteria would be the major source of such BGCs. The latter is probably due to the fact that actinomycetes’ genomes have a high GC content (usually around 70%), and a specific codon bias that would increase the probability of gene expression if a BGC is transferred from a related species. It is also worth mentioning that some *Streptomyces* spp. harbor giant linear plasmids (GLPs) that are self-transmissible, frequently carry several BGCs (Kinashi, 2011) and thus can be involved in the transfer of these BGCs to other bacteria, in particular actinomycetes.

Comparative genomics provides excellent opportunities for tracing evolution of the species, and is an invaluable approach to understanding the spread and functional significance of BGCs. It can pinpoint the differences between strains of the same species isolated from different habitats, which are often in the form of so-called “genomic islands” (GIs) (Penn et al., 2009). Apparently, the GIs represent DNA regions acquired from other species in the process of horizontal gene transfer (HGT), and they often contain remains of mobile genetic elements that most likely have been involved in HGT. Such elements may be represented by transposons, often also hosted by GLPs, and many BGCs contain transposon-like elements, or even look like composite transposons themselves, as in the case of the enterocin biosynthetic gene cluster in marine sponge-derived *Streptomyces albus* (Ian et al., 2014). Recently, Jackson et al. reported the analyses of 13 genomes of streptomycetes isolated from various marine sponges (Jackson et al., 2018). Many unique BGCs were identified, suggesting potential of these bacteria in terms of production of novel bioactive secondary metabolites.

Microbial communities associated with marine sponges, which provide relatively enclosed environment, are especially interesting in terms of tracing the HGT events. In the current work, draft genome sequences for 10 phylogenetically diverse *Streptomyces* spp. isolated from the sponge *Antho dichotoma* collected in the Trondheim fjord (Norway) were obtained. Comparative genomics revealed several of them being closely related to terrestrial *Streptomyces*, and BGC analysis allowed identification of the gene clusters that most likely have been subjects to HGT between the isolates. Several of the isolates exhibited antimicrobial activities, and for two of them bioactive compounds, as well as the BGCs specifying their biosynthesis, were identified.

## Materials and Methods

### Sampling and Treatment of Sponge Samples

Sample of marine sponge *A. dichotoma* was collected from Tautra underwater ridge (specific coordinates 63o 36′ 53″ N, 10o 31′ 22″ E) at the depth of 60 m with the aid of a remote operated underwater vehicle equipped with robotic arm. The sample was immediately transferred to a sterile plastic contained filled with sterilized sea water, and transported to the laboratory within 3 h. Sponge piece of ca 2 cm^3^ was cut out with a sterile scalpel and transferred to a mortar containing 18 mL of 20% glycerol in sea water. The sample was grinded with a pester for ca 2 min, transferred to a 50 mL plastic tube containing 5 g of 3 mm glass beads, and vortexed for 2 min. 8 mL of the resulting suspension was transferred to a sterile plastic tube, and serial dilutions were plated on various agar media as previously described (Ian et al., 2014). The plates were incubated at 20°C for up to 6 weeks.

### Bacterial Strains and Growth Conditions

*Streptomyces* sp. were maintained on ISP4 agar medium (Difco) and soy flour medium (SFM) (Kieser et al., 2000) with followed preparation of spore suspensions in 20% glycerol. Influence of sea water on the growth of *Streptomyces* isolates was tested using ISP2 agar medium (Difco) prepared with and without artificial sea water. The composition of the artificial sea water was as follows (g/L): NaCl, 26.29; KCl, 0.74; CaCl_2_, 0.99; MgCl_2_⋅6H_2_O, 6.09; MgSO_4_⋅7H_2_O, 3.94. *Escherichia coli* K12 and *Bacillus subtilis* 168 (received from the Norwegian University of Science and Technology, Norway) were grown in liquid and soil LB-medium, while *Saccharomyces cerevisiae* BY4743 (received from the Joint Bioenergy Institute, United States) was grown on YPD (Sigma) medium. *Streptomyces* strains and *Sa. cerevisiae* were grown at 30°C, *E. coli* and *B. subtilis* at 37°C.

### Cultivation, Extracts Preparation, and Bioassays

All ten *Streptomyces* strains were cultivated in PM4-1, 5333 and 5280 media (see below for recipes) and their extracts were tested on antimicrobial activity. For preparation of an overnight culture, 20 ml of TSB medium [Oxoid Tryptone Soya Broth powder (CM129) 30 g/L] was inoculated with 50 μl of *Streptomyces* spore suspension and cultivated for 18–20 h in 250 ml baffled Erlenmeyer flasks by 220 rpm. Next day the main culture was prepared by inoculation of 50 ml of test media with 5% of overnight TSB-culture. Test media were: PM4-1: glucose (15 g/L), soy meal (15 g/L), corn steep solids (Sigma, 5 g/L), CaCO_3_ (2 g/L), and 6 ml/L Trace Elements Solution (mg/mL): FeSO_4_⋅7H_2_O, 5.0; CuSO_4_⋅5H_2_O, 0.39; ZnSO_4_⋅7H_2_O, 0.44; MnSO_4_⋅H_2_O, 0.15; Na_2_MoO_4_⋅2H_2_O, 0.01; CoCl_2_⋅6H_2_O, 0.02; HCl, 50; 5333-medium: yeast extract (4 g/L), soluble starch (15 g/L), K_2_HPO_4_ (1 g/L), FeSO_4_⋅7H_2_O (0.5 g/L), pH (7.8); 5280-medium: glycerol (30 g/L), NaCl (5 g/L), CaCO_3_ (1 g/L), pH (7.8).

For the extract preparation 3 mL of culture after 3, 5, and 7 days of cultivation were extracted with 1 ml of butanol and organic phases were tested for the presence of antimicrobial activity against *E. coli* K12, *B. subtilis* 168 and *Sa. cerevisiae* BY4743. The sensitivity of the selected bacteria to extracts was tested by the agar diffusion method by applying 15 μl of extracts on 6 mm filter discs (Whatman) and measuring of growth-inhibition zones.

### DNA Isolation, PCR, and Phylogenetic Analysis

Genomic DNAs were extracted from cultures grown in liquid 3% TSB medium (Oxoid, United Kingdom) at 28°C for 24–48 h using the Qiagen DNeasy Blood and Tissue Kit (QIAGEN, Germany). The 16S rRNA gene fragments were amplified by PCR using the universal bacterial 16S rDNA primers F27 and R1492 (Lane, 1991). Obtained PCR products were purified and directly sequenced with the F27 and R1492 primers at EUROFINS (Germany). DNA sequences of almost complete 16S rRNA genes (ca 1400 nucleotides) were compared with those in the GenBank. Phylogenetic tree based on these sequences along with those of the validly named species retrieved using Ez BioCloud^1^ as most similar was constructed using the Molecular Evolutionary Genetics Analysis (MEGA) software version 7 (Kumar et al., 2016). The tree was computed using the Neighbor-Joining method, and the resulting tree topology was tested by bootstrap analysis performed with 500 replicates.

### Genome Sequencing, Assembly, and Analyses

For genome sequencing of each sample, two WGS sequencing libraries were prepared, using the TruSeq DNA PCR-Free Kit respectively the Nextera Mate Pair Library Preparation Kit (Illumina Inc., San Diego, CA, United States) according to the manufacturer’s instructions. The resulting libraries were sequenced on the MiSeq sequencing platform using the MiSeq Reagent Kit v3 in 2 × 300 nt sequencing runs. The sequencing data sets were assembled using the Newbler assembler v2.8 (454 Life Sciences, Branford, CT, United States). In case of ADI95-16, the initial assembly was then manually curated in Consed (Gordon and Green, 2013), resulting in a single contig per replicon for each, the linear chromosome and the four plasmids (three of them linear, one circular). The assembled contigs were annotated using the Prokka pipeline v1.11 (Seemann, 2014), including the annotation of non-coding RNAs. The relevant data for the assembly and genome annotation of the ten strains are listed in Supplementary Table S1.

The Whole Genome Shotgun projects have been deposited at DDBJ/ENA/GenBank under the accessions RPGS00000000 (ADI91-18), RPGT00000000 (ADI92-24), RPGU00000000 (ADI93-02), CP033581-CP033585 (ADI95-16), RPGV00000000 (ADI95-17), RPGW00000000 (ADI96-02), RPGX00000000 (ADI96-15), RPGY00000000 (ADI97-07), RPGZ00000000 (ADI98-10), and RPHA00000000 (ADI98-12). The versions described in this paper are version 1 (XXXX01000000).

Analysis of the genomes for secondary metabolite BGCs was done with antiSMASH 5.0 online tool (Blin et al., 2019), and the identified clusters and their genes involved in scaffold assembly were queried against the MiBIG database (Medema et al., 2015).

### Construction of the ADI95-16 Knock-Out Mutant

The DNA fragment (0.99 kb), containing the central part of *ftdB* gene identified in the polycyclic tetramate macrolactam (PTM) cluster was amplified by PCR from gDNA of *Streptomyces* spp. ADI95-16; the primers 95-16_C22ko_F and 95-16_C22ko_R were used. These primers included the restriction sites *Eco*RI and *Hin*dIII respectively, to allow proper ligation with the 3.0 kb *Eco*RI-*Hin*dIII fragment from pSOK201 plasmid containing ColE1, *oriT* and Am^*R*^. The resulting construct pPTM95-16KN was introduced in *Streptomyces* sp. ADI95-16 via conjugation from *E. coli* ET12567/pUZ8002. Apramycin (50 μg/mL) was used for selection of recombinant *Streptomyces* strains. Oligonucleotide primers were designed using Clone Manager 9 software (Sci-Ed Software, United States).

### Production, Purification, and Identification of Linearmycins and Echinomycins

A 10 mL of TSB medium (Oxoid, United Kingdom) was inoculated with 200 μL of a dense spore suspension of *Streptomyces* spp. ADI95-16 in a 100 mL Erlenmeyer flask and incubated at 28°C with 200 rpm over night to produce seed culture. For the purification of linearmycins, ten 250 mL baffled Erlenmeyer flasks containing each 50 mL of ikarugamycin production medium (starch 2%, cotton seed flour 2%, corn steep liquor 1% and CaCO_3_ 0.3%, in 1 L of distilled water, pH 6.2) were inoculated with 3 mL of seed culture and fermented at 28°C with 200 rpm for 48 h. The whole culture (0.5 L) was freeze-dried and extracted with 250 mL mixture of chloroform and methanol (1:1) during 2 h. The organic phase was evaporated *in vacuo* to generate a dry layer that was suspended in methanol to generate a concentrated extract. The methanolic crude extract was separated using a Shim-pack GIS C18, 250 mm × 20 mm, 5 μm column (Shimadzu) in a Semi-preparative HPLC Shimadzu LC-20AR equipment. 0.1% aqueous formic acid and acetonitrile were used as mobile phase A and B, respectively. The gradient used was: 5–95% B in 45 min followed by a washing (10 min at 95% B) and re-equilibration step (10 min at 10% B), flow rate was 20 mL/min and 190 nm wave length was used for detection. Linearmycin A eluted with a retention time of 21 min, and linearmycin B with a retention time of 22 min. Approximately 3.0 mg of linearmycin A and 2.5 mg of linearmycin B were obtained.

For the production of echinomycins, 10 mL of TSB medium (Oxoid, United Kingdom) was inoculated with 200 μL of a dense spore suspension of *Streptomyces* spp. ADI96-02 in a 100 mL Erlenmeyer flask and incubated at 28°C with 200 rpm over night to produce seed culture. Then, a 250 mL baffled flask containing 50 mL of MYM medium (4 g/L maltose, 4 g/L yeast extract, 10 g/L malt extract, 1.9 g/L MOPS) was inoculated with 3 mL of seeding culture. The fermentation was carried out at 200 rpm and 28°C for 7 days. Fermentation culture was harvested and freeze dried. 25 mL of methanol was used to extract the dried material at 200 rpm and room temperature for 1 h. The methanolic extract was concentrated *in vacuo* and analyzed by LC-MS/MS.

LC-MS analyses were performed on two different instruments. All extracts were analyzed on an UltiMate 3000 series system HPLC equipped with a VWD detector (Dionex/Thermo Fisher Scientific, Germering, Germany) that was coupled to a maXis UHR ESI-Qq-TOF mass spectrometer (Bruker Daltonics, Bremen, Germany). Separation was carried out on an Acclaim 120 C18, 2.1 × 150 mm, 3 μm HPLC column (Thermo Fisher Scientific) using water and acetonitrile, both modified with 0.1% formic acid, as mobile phase A and B, respectively. The sample components were separated and eluted with a linear gradient from 5 to 95% B in 45 min followed by an isocratic column cleaning (9.5 min at 95% B) and re-equilibration step (10 min at 5% B). The flow rate was 0.45 mL/min and the column oven temperature was set to 25°C. After passing the UV detector, high-resolution MS and MS/MS spectra were recorded in positive ion mode in the range *m/z* 50–2000. The following ESI ion source settings were applied: capillary voltage: 4.5 kV, nebulizer: 1.2 bar (N_2_), dry gas flow: 8.0 L/min (N_2_), and dry temperature: 180°C. MS/MS spectra were obtained in automated data-dependent acquisition mode using argon as collision gas, an isolation window of Δ*m/z* = 4, and an *m/z*- and charge-dependent fragmentation amplitude. In the case of echinomycin and echinoserine, targeted MS/MS experiments were performed using an isolation window of Δ*m/z* = 6 and a fragmentation amplitude of 30 eV to facilitate comparison with literature data (Williamson et al., 1982). The sum formulas of the detected ions were determined using Bruker Compass DataAnalysis 4.0 based on the mass accuracy (Δ*m/z* ≤ 5 ppm) and isotopic pattern matching (SmartFormula algorithm).

Echinomycin (≥98% HPLC) was obtained from Sigma-Aldrich (St. Louis, MO). LC-MS measurements of the ADI96-02 extract, the echinomycin reference standard, and the ADI96-02 extract spiked with the echinomycin reference standard for comparison of the retention time as well as the positive and negative ion mode MS and MS/MS spectra were conducted on a Vanquish Horizon UHPLC system (Thermo Fisher Scientific) coupled to the ESI source of an LTQ Orbitrap Velos mass spectrometer (Thermo Fisher Scientific).

## Results

### Isolation and Preliminary Characterization of *Streptomyces* spp. From the Marine Sponge *Antho dichotoma*

**FIGURE 1:**
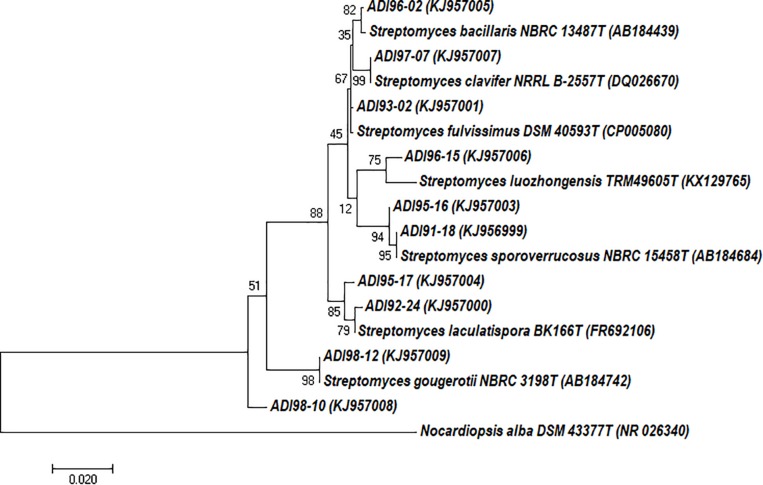
Evolutionary relationships of *Streptomyces* spp. isolated from *Antho dichotoma* based on the 16S rRNA gene sequences. The evolutionary history was inferred by using the Maximum Likelihood method. The tree is drawn to scale, with branch lengths measured in the number of substitutions per site. The analysis involved 18 nucleotide sequences. Evolutionary analyses were conducted in MEGA7 (35).

A sample of the marine sponge collected at the Tautra underwater ridge in the Trondheim fjord (Norway) and classified as *A. dichotoma* was used to isolate actinomycete bacteria (see Materials and Methods). In total, 497 isolates were recovered from different agar media, macromorphology of which suggested them being actinobacteria. One hundred and eight of these isolates were tested for molecular phylogeny using 16S rRNA gene amplicons. Seventy nine of the tested isolates were classified as *Streptomyces*, while the rest was representated by actinobacterial genera *Actinoalloteichus*, *Micromonospora*, *Actinomadura*, *Promicromonospora*, *Isoptericola*, *Rhodococcus*, *Nocardiopsis*, and *Pseudonocardia* (data not shown). Dereplication of the 79 *Streptomyces* isolates based on the 16S rRNA gene fragment sequences (1350–1475 nt) yielded 51 isolates that were considered diverse (Supplementary Figure S1). For this study, we selected 10 *Streptomyces* spp. isolates that were both morphologically (visual inspection) and phylogenetically diverse (Figure 1). The 16S rRNA gene sequence identities between the selected isolates and their closest validated phylogenetic neighbors were in the range of 98–99%.

These *Streptomyces* isolates were first tested for growth on ISP2 medium with and without sea water. Apparently, the growth and morphological differentiation of nearly all strains was affected by sea water, except for the isolate ADI92-24, which grew equally well on both media (Table 1). In order to test whether we can detect the production of antimicrobial compounds in these isolates, all 10 strains were grown in 3 different liquid media (see Materials and Methods) which generally support production of secondary metabolites (unpublished data). The *n*-butanol extracts prepared by liquid-liquid extraction of the cultures sampled after 3, 5, and 7 days of fermentation were used in bioassays against *B. subtilis*, *E. coli*, and *Sa. cerevisiae* (results are shown in Supplementary Table S1). Rather surprisingly, and in contrast to what has been observed for *Streptomyces* species isolated from terrestrial sources, very limited bioactivity was revealed in these tests. Only 4 out of 10 isolates showed antibiotic activity in the conditions tested. Considering the fact that *Streptomyces* in general harbor >20 gene clusters for the biosynthesis of secondary metabolites, some of which usually have antibiotic activity, the latter result suggested that media and conditions used were not sufficient to induce biosynthesis of secondary metabolites in most of these isolates.

**TABLE 1:** Effect of sea water on the growth of sponge-derived *Streptomyces* spp. on ISP2 medium.

***Streptomyces* isolate**	**ISP2**	**ISP2 + sea water**
ADI91-18	+	–
ADI92-24	–	–
ADI93-02	–	+
ADI95-16	+	–
ADI95-17	–	+
ADI96-02	+	–
ADI96-15	+	–
ADI97-07	–	+
ADI98-10	–	+
ADI98-12	–	+

*(+) indicates faster growth and morphological differentiation; (–) no effect (by comparison).*

### Comparative Genomics of Sponge-Associated *Streptomyces* Focused on Secondary Metabolite Biosynthesis Gene Clusters

In order to obtain an insight into the secondary metabolite biosynthesis potential of the isolates, and to detect any possible transfer of BGCs between them, draft genome sequences were obtained for all strains. Remarkably, genomes of several sponge isolates showed considerable synteny with the genomes of some terrestrial streptomycetes. In particular, *Streptomyces* sp. ADI98-12 vs. *S. albus* J1074, *Streptomyces* sp. ADI95-16 and ADI91-18 vs. *Streptomyces* sp. Mg1, and *Streptomyces* sp. ADI96-02 vs. *Streptomyces fulvissimus* DSM 40593 (Supplementary Figures S2–S5). The draft genomes were then analyzed for the presence of BGCs using antiSMASH 5.0 online software (Blin et al., 2019). Data obtained with antiSMASH were manually curated to ensure optimized prediction of the gene cluster borders, since the software occasionally considered two adjacent but clearly distinct BGCs as one. In particular, product of each gene was queried against GenBank using BLASTp search engine, and gene encoding proteins without any obvious role in secondary metabolism were considered as flanking the cluster borders. Also, corrections were made when one cluster was split between two or more contigs. A summary of the results of this analysis is presented in Table 2, and particular genome features are given in Supplementary Table S2. According to this analyses, isolate ADI95-17 has the largest genome of ca 10 Mb which encodes at least 36 BGCs most of which are represented by PKS- and NRPS-type clusters. Isolate ADI96-02 possessed the smallest ca 7 Mb genome among all isolates, which still encoded at least 30 BGCs most of which were represented by RiPPs, terpenoid, and hybrid PKS-NRPS clusters. The largest number of BGSs, was identified in the 9.1 Mb genome of isolate ADI95-16. The majority of these BGCs were represented by PKS, RiPPs and terpenoid clusters. The rest of the isolates had genome sizes that ranged from 7.1 to 9.3 Mb, and harbored between 28 and 34 BGCs per genome.

**TABLE 2:** Secondary metabolite biosynthesis gene clusters in the genomes of sponge-derived *Streptomyces* spp.

**Strain**	**Genome size, Mb**	**PKS I, II, III**	**NRPS**	**PKS/NRPS**	**RiPP**	**Terp**	**Siderophore**	**Other**	**Total**
ADI91-18	8.64	9	2	3	7	7	3	5	34
ADI92-24	9.31	6	5	3	1	8	1	4	28
ADI93-02	8.41	4	3	2	2	6	2	10	29
ADI95-16	9.11	8	3	3	8	7	3	7	39
ADI95-17	10.03	9	7	3	2	5	1	9	36
ADI96-02	6.99	4	3	5	5	6	2	5	30
ADI96-15	7.08	7	6	4	3	5	2	3	30
ADI97-07	8.35	8	3	5	3	4	3	7	33
ADI98-10	7.72	4	3	2	5	6	2	6	28
ADI98-12	7.24	3	6	6	3	4	2	5	29

*The analysis was done using antiSMASH 5.0 online software with subsequent manual curation.*

As expected from the high degree of synteny of the draft sequences to genomes of terrestrial *Streptomyces* species, the draft genomes of the sponge-associated streptomycetes were fairly typical, with sequence lengths between 6.99 Mbp and 10.03 Mbp and G + C contents between 70.63 and 73.42%. Likewise, the automated assemblies were also rather typical for MatePair-guided Illumina-only assemblies, with the drafts consisting of 2–18 scaffolds containing 32–377 contigs. Due to the low number of scaffolds and contigs, the genome of ADI95-16 could be finished manually, resulting in just one scaffold/contig for each of the 5 replicons. Finally, the automated annotation revealed some differences concerning the number of ncRNAs detected by INFERNAL (Nawrocki and Eddy, 2013) against the RFAM database (Kalvari et al., 2018), which ranged from 3 up to 33 for the 10 isolates investigated. Closer inspection of the predicted ncRNAs revealed that this large variation is due to the number of predicted ASpks and ASdes sRNAs, which range from 1 up to 30. “ASdes TB sRNA” and “ASpks TB sRNA” are two ncRNAs that were initially identified in *Mycobacterium tuberculosis* in the *desA1* and *pks12* genes, respectively. Gene *desA1* encodes an acyl-acyl carrier protein desaturase that catalyzes the introduction of specific double bonds during the biosynthesis of mycolic acids in Mycobacteria, while the *pks12* gene encodes an PKS enzyme responsible for the biosynthesis of lipid moiety of the mycobacterial phospholipid antigen. Similar ncRNAs are found in many *Streptomyces* spp., and are believed to be involved in gene regulation, in particular regulation of BGC expression (Arnvig and Young, 2009).

Some of the BGCs identified in the genomes of the sponge-associated streptomycetes could be linked to already known molecules based on their high similarity to already characterized BGCs (identical gene composition and >75% identity of the gene products) and was annotated in MiBIG database (Medema et al., 2015). Beside BGCs for ectoine, desferrioxamine B, geosmin, 2-methylisoborneol, isorenieratene, melanin, hopanoids, albaflavenone, and spore pigment, commonly present in the streptomycete genomes, the BGCs for less abundant secondary metabolites were also identified (Table 2). In addition to the abovementioned BGCs, many of the predicted not-characterized BGCs were unique for each isolate, albeit sometimes being present in the genomes of their terrestrial counterparts or other *Streptomyces* spp. deposited in the public databases. The latter may reflect both different evolutionary origins of the isolates, as well as the timing of their acquisition by the sponge and hence different exposure to the HGT events. However, a number of gene clusters appeared to be either well conserved among certain isolates, suggesting vertical gene transfer (VGT), or might have been recently transmitted via HGT. In particular, the HGT hypothesis concerned gene clusters for lantipeptide and thiopeptide biosynthesis, while VGT could be implied for the gene clusters specifying tetramate macrolactams identified in 8 out of 10 isolates (see below).

### Gene Clusters for Lantipeptide and Thiopeptide Biosynthesis

**FIGURE 2:**
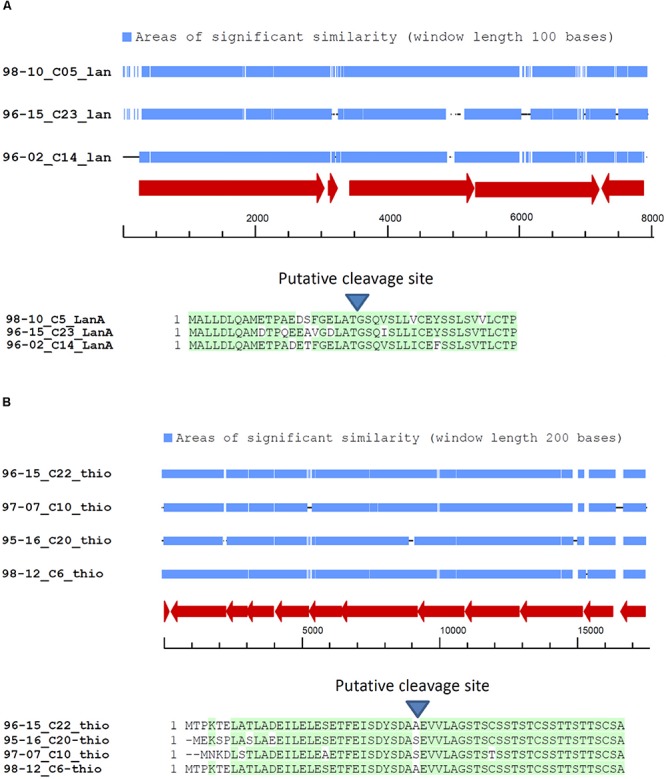
BGCs in the genomes of sponge-derived *Streptomyces* spp. presumed to be subjects to horizontal gene transfer. **(A)** Alignment of the lantipeptide BGCs and the encoded pre-propeptide sequences. **(B)** Alignment of the thiopeptide BGCs and the encoded pre-propeptide sequences.

In total, 30 BGCs for lantipeptide biosynthesis were identified in the genomes of 10 sponge-derived *Streptomyces* isolates. Comparison of the amino acid sequences of the lantipeptide precursors revealed striking similarities between some BGCs present in the genomes of different isolates. In particular, similarities between both pre-propeptides and gene organization of a particular cluster were evident in isolates ADI96-02, ADI96-15, and ADI98-10 (Figure 2A). Alignment and closer examination of nucleotide sequences of these clusters confirmed their high similarity (70–75% identity), while most differences could be seen in the regulatory regions, especially upstream of the gene encoding the lantipeptide precursor (Figure 2A). A similar observation was made for one thiopeptide BGC identified in the isolates ADI96-15, ADI95-16, ADI97-07, and ADI98-12, where the amino acid sequences of pre-propeptides shared even greater homology (Figure 2B). Although no apparent direct or inverted repeats could be identified within the DNA regions flanking the abovementioned BGCs, and there were no transposase genes in their vicinity, it seems possible that these clusters have been subjects to a relatively recent HGT. This suggestion is further supported by the phylogenetic diversity of the isolates where highly similar BGCs were found, which makes it less probable that these clusters have been retained due to the VGT. In the process of evolution, their sequences may have changed and diversified as a result of adaptation to the new host’s cellular environment and regulatory networks.

### Gene Clusters for Polycyclic Tetramate Macrolactam Biosynthesis

**FIGURE 3:**
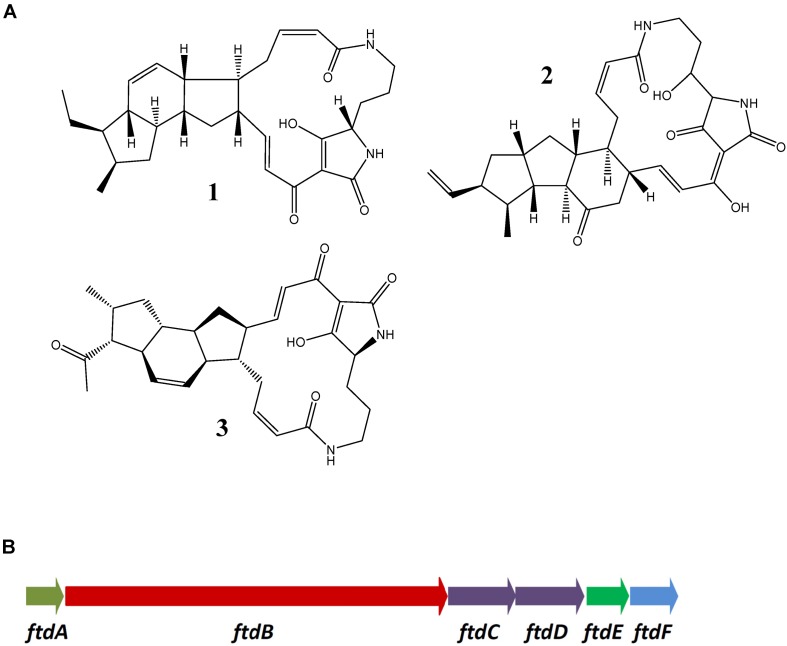
Polycyclic tetramate macrolactams from streptomycetes. **(A)** Ikarugamycin (1), frontalamide B (2), and clifednamide (3). **(B)** BGC for frontalamides from *Streptomyces* sp. SPB78 [17].

Recently, biosynthesis of PTM antibiotics, some of which have antifungal activity, received considerable attention. Biosynthesis of several PTMs was studied in some detail, including heat-stable antifungal factor (HSAF) from *Lysobacter enzymogenes* (Yu et al., 2007), as well as ikarugamycin, frontalamides, and clifednamides from *Streptomyces* spp. (Cao et al., 2010). Chemical structures of these antifungal antibiotics are shown on Figure 3A. The BGC for frontalamide (Figure 3B) represents a typical PTM biosynthetic gene cluster identified in various *Streptomyces* spp. (Blodgett et al., 2010). The frontalamide BGC encodes a fatty acid hydroxylase FtdA, a hybrid PKS-NRPS FtdB, desaturases FtdC, and FtdD, zinc-dependent dehydrogenase FtdE and P450 monooxygenase FtdF. Precursors for the polycyclic mactrolactam ring, which can vary in size, are synthesized by an iterative hybrid PKS-NRPS, such as FtdB, which utilizes malonyl-CoA and ornithine as substrates. The hybrid chain is cyclized via the action of two enzymes belonging the phytoene desaturase family, i.e., Ftd C and FtdD. The fatty acid hydroxylase FtdA installs a hydroxyl group on the macrolactam ring. Some PTM clusters, notably from non-streptomycete bacteria, lack *ftdE* and *ftdF* homologs, which further diversify the PTM structures via changes in the oxidation pattern (Blodgett et al., 2010).

Apparently, BGCs for PTMs are wide-spread in the bacterial kingdom, suggesting that the metabolites they specify play an important role in the environmental adaptation (Blodgett et al., 2010). Analysis of the genomes of *Streptomyces* pp. isolated from *A. dichotoma* revealed the presence of PTM BGCs in 8 out of 10 isolates. Six out of eight PTM BGCs identified in the isolates ADI92-24, ADI95-17, ADI96-02, ADI96-15, and ADI98-12 had a gene composition and arrangement identical to that of the frontalamide BGC. The PTM BGCs from ADI91-18 and ADI95-16 (virtually identical on nucleotide level) appeared different in that they lacked the *ftdE* gene homologs. The latter implied that these clusters may encode a novel PTM with unique oxidation pattern. An attempt was made to identify the product of the PTM BGC in isolate ADI95-16, for which a gene transfer system based on intergenic conjugation was established. The strategy for identification was based on construction of a knock-out mutant of the *ftdB* homolog encoding hybrid PKS-NRPS in the PTM cluster, followed by comparison of metabolite profiles of the mutant and the wild type strain. A 0.99 kb DNA fragment representing the central part of this gene was PCR-amplified from the genomic DNA of ADI95-16 and ligated with the part of pSOK201 (Zotchev et al., 2000), generating a suicide knock-out vector pPTM95-16KN. Upon conjugative transfer of this vector to ADI95-16, one knock-out mutant was obtained. Comparative analyses of metabolites produced by the ADI95-16 and its PTM knock-out mutant cultivated in different media using LC-MS/MS did not reveal any relevant differences, suggesting that the cluster is most likely not expressed and the polycyclic macrolactam is not produced under the conditions tested.

### Bioactivity of Some Sponge-Derived Streptomycetes Is Due to Production of Linearmycins, Cycloheximide, and Echinomycins

Since isolates ADI95-16 and ADI96-02 displayed good bioactivity against *B. subtilis* and *Sa. cerevisiae*, we decided to identify the compounds responsible for these activities, and to connect the identified molecules with the respective BGCs. Isolate ADI95-16 was cultivated in ikarugamycin production medium (Jomon et al., 1972), and extracts were subjected to bioactivity-guided fractionation that resulted in purification of bioactive compounds. This was followed by the analyses of the bioactive extracts by means of LC-MS/MS. The comparative genome analysis of ADI95-16 resulted in the detection of a BGC for a polyene polyketide by the antiSMASH software, which was at first erroneously annotated as the one for ECO-02301 (McAlpine et al., 2005). Indeed, HPLC and LC-MS analysis of the bioactive CHCl_3_/MeOH extract showed several main compounds whose UV and mass spectra pointed to this compound class (Supplementary Figures S6–S10). A search of the Dictionary of Natural Products and comparison of the obtained MS/MS spectra with literature data identified the two main congeners as linearmycin A and B, antibiotics similar to ECO-02301 that were previously reported from *Streptomyces* sp. Mg1 (Hoefler et al., 2017). The genome of *Streptomyces* sp. Mg1 shares high degree of similarity with that of ADI95-16 and comparison of the complete linearmycin gene clusters from ADI95-16 and Mg1 revealed 96% identity on nucleotide level (data not shown). Interestingly, while the two major linearmycin congeners looked identical between the two strains, the profile of the minor congeners was different. In particular, we did not detect the previously reported linearmycin C (Hoefler et al., 2017), but instead found a derivative with the sum formula C_71_H_113_NO_21_ (HRESIMS *m/z* 1316.7882 [M + H]^+^; calculatedd for C_71_H_114_NO_21_^+^, *m/z* 1316.7878, Δ = 0.3 ppm) with similar intensity as linearmycin B (Supplementary Figures S7, S8). The fragmentation pattern indicates that a C_7_H_12_O_5_ group, most likely a methylhexosyl moiety, is attached to the amine group of linearmycin A (Supplementary Figures S9, S10). The corresponding derivative of linearmycin B was also detected with lower abundance.

**FIGURE 4:**
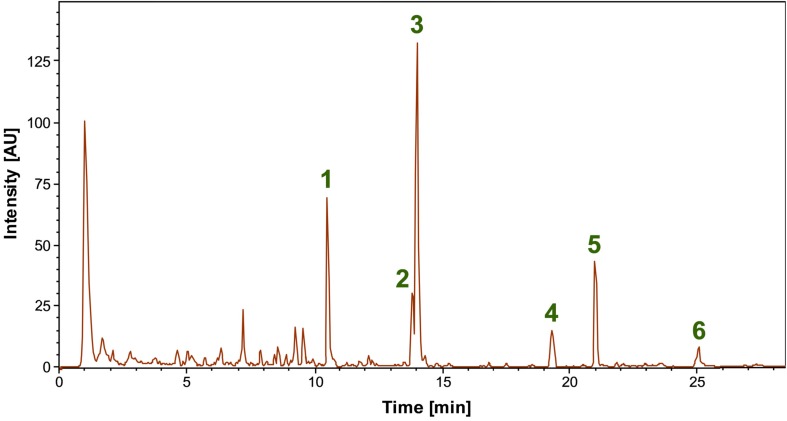
Base peak chromatogram of the bioactive methanolic extract of *Streptomyces* spp. ADI96-02 in the range *m/z* 50-2000. Several main compounds were tentatively identified as nocardamine **(1)**, cycloheximide **(3)**, an isomer of cycloheximide **(2)**, actiphenol **(5)**, echinoserine **(4)**, and echinomycin **(6)**, with the identity of the latter being confirmed by standard addition.

LC-MS analyses of the bioactive methanolic extract of ADI96-02 obtained after strain cultivation in the MYM medium lead to the identification of several main constituents (Figure 4), most of them matching the predictions for bioactive compounds by the antiSMASH-based genome analyses (Table 2). The highest peak in the base peak chromatogram (**3**) was tentatively assigned to cycloheximide based on the accurate mass (HRESIMS *m/z* 282.1705 [M + H]^+^; calcd for C_15_H_24_NO_4_^+^, *m/z* 282.1700, Δ = 1.9 ppm) and a very high similarity of the MS/MS spectrum (Supplementary Figure S11) to reference data in the mzCloud database.^2^ A less abundant compound with identical mass and fragment ions, most likely an isomer of cycloheximide such as isocycloheximide or naramycin B (**2**), and the related compound actiphenol (**5**) were also detected (Supplementary Figures S12, S13) (Yin et al., 2014; Grubbs et al., 2019).

Another main compound (**1**) detected in ADI 96-02 was tentatively identified as the siderophore nocardamine (deferrioxamine E), again by accurate mass (HRESIMS *m/z* 601.3533 [M + H]^+^; calcd for C_27_H_49_N_6_O_9_^+^, *m/z* 601.3556, Δ = −3.7 ppm) and comparison of the MS/MS spectrum (Supplementary Figure S14) to reference data in mzCloud^3^ and in literature (Novák et al., 2017). Finally, two peaks could be assigned to the predicted non-ribosomal peptides echinoserine (**4**) and echinomycin (**6**). Echinoserine (HRESIMS *m/z* 1137.4522 [M + H]^+^; calculated for C_51_H_69_N_12_O_14_S_2_^+^, *m/z* 1137.4492, Δ = 2.6 ppm), which is the non-cyclic form of echinomycin first described in *Streptomyces tendae*, was confirmed with very high certainty by an excellent match of the characteristic MS/MS spectra (Supplementary Figure S15) with literature data (Blum et al., 1995; Socha et al., 2009). Despite being the much better investigated congener, no high quality reference MS/MS spectra could be found for echinomycin (HRESIMS *m/z* 1101.4303 [M + H]^+^; calcd for C_51_H_65_N_12_O_12_S_2_^+^, *m/z* 1101.4281, Δ = 2.2 ppm). Thus, the production of echinomycin in ADI96-02 was unambiguously confirmed by comparison with a commercially available reference standard followed by LC-MS analysis (Supplementary Figures S16, S17). Echinomycin is a cytotoxic compound previously reported from *Streptomyces echinatus* (Williamson et al., 1982). Later, the echinomycin BGC from *Streptomyces lasaliensis* was identified and characterized (Watanabe et al., 2006). Interestingly, the echinomycin BGC in ADI96-02 harbored a gene encoding a putative monooxygenase, which was replaced by a transposase in the similar BGC of *S. lasaliensis*. Also, the ADI96-02 cluster contained 2 genes for ABC transporters that were absent on the *S. lasaliensis* counterpart (data not shown).

Additional data on the fragmentation patterns obtained with MS/MS analyses and confirming the identity of linearmycins, echinomycin, echinoserin, cycloheximide, actiphenol, and nocardamine are given in Supplementary Figures S18–S26.

### Comparative Genomics of Marine *Streptomyces* sp. ADI95-16 and Terrestrial *Streptomyces* sp. Mg1

**FIGURE 5:**
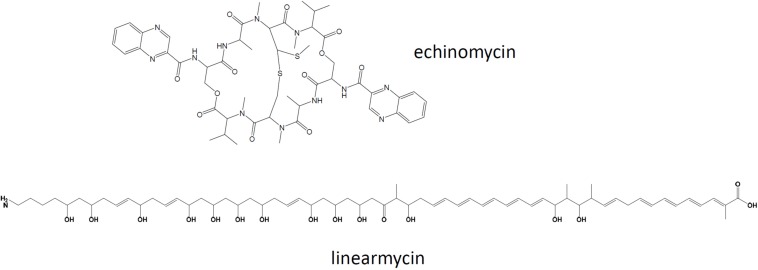
Chemical structures of linearmycin A and echinomycin shown to be produced by *Streptomyces* sp. ADI95-16 and ADI96-02, respectively.

Due to the high similarity of the genomes of the sponge isolate *Streptomyces* sp. ADI95-16 and the soil-derived *Streptomyces* sp. Mg1 suggested by synteny of draft genomes, it appeared interesting to compare the secondary metabolite biosynthesis potential of these strains. The genomes’ similarity was strongly supported by the DDH value calculated using GGDC tool (Meier-Kolthoff et al., 2013), which ranged from 73.2 to 81.0, depending on the calculation formula used. To achieve a full-scale comparison, the genome of ADI95-16 was completely sequenced, revealing a linear chromosome of ca 8.2 Mb, three linear plasmids of 630 kb, 255 kb, 10.7 kb and one circular plasmid of 7 kb. *Streptomyces* sp. Mg1 possessed a linear chromosome of ca 7.9 Mb, two linear plasmids of 530 kb and 184 kb, and one circular plasmid of 135 kb (Hoefler et al., 2013). The latter strain has been reported to produce linearmycins (Figure 5), polyketides with antifungal and antibacterial activities (Stubbendieck et al., 2018).

**FIGURE 6:**
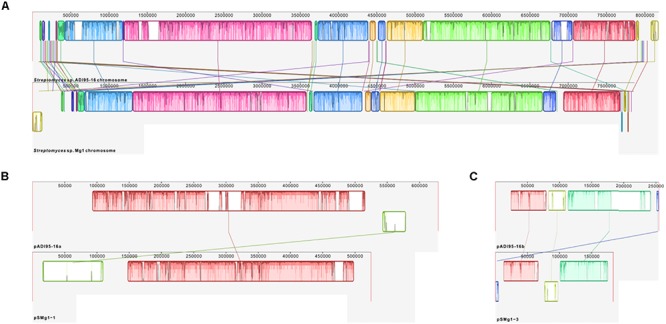
Genomic synteny of *Streptomyces* spp. ADI95-16 and Mg1, based on a progressive alignment with MAUVE (Darling et al., 2010). **(A)** Chromosomes. **(B)** pADI95-16a (reversed) versus pSMg1-1. **(C)** pADI95-16b (reversed) versus pSMg1-3.

**FIGURE 7:**
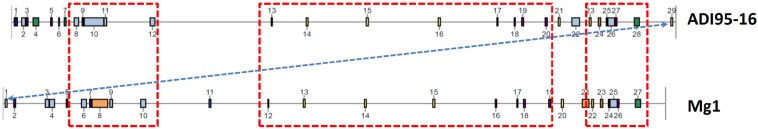
Localization of BGCs on the chromosomes of *Streptomyces* spp. ADI95-16 and Mg1. Largest homologous chromosomal regions harboring identical BGCs are boxed with red dotted lines, and translocation of the nucleoside BGC is indicated by a dotted blue arrow.

Synteny of the chromosomes and the two largest linear plasmids from the two strains, all of which, according to the antiSMASH analysis, encode secondary metabolite BGCs, are shown in Figure 6. Next, we examined the BGCs detected upon antiSMASH analysis followed by manual curation in the genomes of *Streptomyces* spp. ADI95-16 and Mg1. Chromosomes of both strains harbored 23 BGCs that were virtually identical and positioned in the same order on the physical map, with the exception of a putative nucleoside BGC that is located at the opposite chromosomal termini in ADI95-16 and Mg1. In comparison with Mg1, the ADI95-16 chromosome contained unique BGCs for terpene, ectoine, cyclic diketopiperazine, lantipeptide, and NRPS-PKS hybrid-class compounds. Mg1, in comparison to the ADI95-16 counterpart, harbored unique BGCs for a carotenoid, avermitilol, a lasso peptide, a lantipeptide, an unknown terpene, and a chalcomycin-related polyketide. The arrangement of the BGCs in the chromosomes of two strains is shown in Figure 7. The largest linear plasmids of both strains, pADI95-16a (630 kb) and pSMg1-1 (530 kb), shared two BGCs for butyrolactones and one for a PKSI-derived polyketide, while pADI95-16a harbored in addition a BGC for a symocyclinone-like compound and a lantipeptide. The smaller linear plasmids of both strains, pADI95-16b (255 kb) and pSMg1-3 (184 kb), contained BGCs for an enedyine, an arylpolyene, and a sporolide-like compound. The latter plasmids differed, however, in that pADI95-16b contained an additional BGC for a butyrolactone, while pSMg1-3 harbored a BGC for a streptonigrin-like compound.

## Discussion

Actinomycete bacteria from underexplored habitats may produce novel bioactive secondary metabolites, which can be developed into much needed drugs to fight infections and cancer. Quite often, as it was revealed in recent studies (Ian et al., 2014), such isolates do not produce many bioactive secondary metabolites under standard laboratory conditions. Indeed, only 4 out of 10 *Streptomyces* isolates obtained from a marine sponge and investigated in this work were found to be bioactive in conditions tested. Hence, their genomes were investigated in order to reveal biosynthetic potential of these isolates. Having information on the BGCs, especially those that may potentially specify biosynthesis of novel compounds, opens possibilities for genome mining that can be applied to trigger gene expression of particular BGCs (Sekurova et al., 2019). Also, comparative genomics can be used to trace transfer of BGCs between isolates, which could be expected to happen considering spatial containment within the sponge.

Out of the 10 *Streptomyces* isolates investigated, 9 differed significantly both in terms of taxonomy, genome sizes and BGCs detected. The exception were isolates ADI95-16 and ADI91-18, which were closely related (Figure 1), shared most of the BGCs (data not shown), and were apparently also related to the terrestrial isolate *Streptomyces* sp. Mg1. Two other isolates, ADI98-12 and ADI96-02 were also found to have terrestrial counterparts. Thanks to the growing database of characterized BGCs (Medema et al., 2015), we were able to unambiguously assign many BGCs in the sequenced genomes to the known ones (Table 3), thus assigning the ability to produce certain secondary metabolites to particular isolates. Together, the 10 *Streptomyces* isolates can potentially produce at least 29 already known secondary metabolites, while judging from their total number of BGCs, this potential exceeds 300 compounds, not counting congeners. This calculation clearly suggests that even this small number of streptomycetes can provide a huge arsenal of bioactive molecules that may benefit their host and protect it from predators and infectious agents.

**TABLE 3:** Potential of the sponge-derived *Streptomyces* spp. to produce known secondary metabolites deduced from the antiSMASH-based genome analyses followed by manual curation.

**Strain**	**Identified BGC**	**Compound class**	**Biological activity**	**References**
ADI91-18	Alkyl-O-dihydrogeranyl-methoxyhydroquinones	Prenylated phenolic lipid	–	Awakawa et al., 2011
	Linearmycins	Polyketide	Antibacterial, antifungal	Stubbendieck et al., 2018
	Avermitilol	Sesquiterpenoid	–	Chou et al., 2010
	SapB peptide	Lantipeptide	Peptidic morphogen	Kodani et al., 2004
ADI92-24	Tomaymycin	Pyrrolobenzodiazepine	Cytotoxic	Li et al., 2009
ADI93-02	Alkyl-O-dihydrogeranyl-methoxyhydroquinones	Prenylated phenolic lipid	–	Awakawa et al., 2011
	Mirubactin	NR peptide	Siderophore	Giessen et al., 2012
	Coelichelin	NR peptide	Siderophore	Challis and Ravel, 2000
	PM100117	Macrolide	Cytotoxic	Salcedo et al., 2016
ADI95-16	Linearmycins	Polyketide	Antibacterial, antifungal	Stubbendieck et al., 2018
	Erythrochelin	Hydroxamate	Siderophore	Robbel et al., 2010
ADI95-17	Mirubactin	NR peptide	Siderophore	Giessen et al., 2012
	Coelichelin	NR peptide	Siderophore	Challis and Ravel, 2000
	Naringenin	Flavonoid	Antioxidant, cytotoxic	Álvarez-Álvarez et al., 2015
ADI96-02	Cycloheximide	Dicarboximide	Antifungal	Yin et al., 2014
	Galbonolides	Macrolide	Antifungal	Kim et al., 2014
	SRO15-3108	Lantipeptide	–	Kersten et al., 2011
	Echinomycin	NR peptide	Cytotoxic	Watanabe et al., 2006
	Alkylresorcinol	Phenolic lipid	–	Funa et al., 2006
	Coelichilin	NR peptide	Siderophore	Challis and Ravel, 2000
ADI96-15	Candicidin	Polyene macrolide	Antifungal	Chen et al., 2003
	Antimycin	NR peptide-polyketide	Cytotoxic	Yan et al., 2012
	Lobophorins	Spirotetronate	Cytotoxic	Yue et al., 2016
	SAL-2242	Lantipeptide	–	Kersten et al., 2011
ADI97-07	Clavams	NR peptide	Antibacterial	Zelyas et al., 2008
	Galbonolides	Macrolide	Antifungal	Kim et al., 2014
	Neocarzilin	Polyketide pyrone	Cytotoxic	Otsuka et al., 2004
	Antimycin	NR peptide-polyketide	Cytotoxic	Yan et al., 2012
	Legonaridin	Linaridin	–	Rateb et al., 2015
	Coelichilin	NR peptide	Siderophore	Challis and Ravel, 2000
ADI98-10	SRO15-2005	Lasso peptide	–	Kersten et al., 2011
	AmfS peptide	Lantipeptide	Peptidic morphogen	Ueda et al., 2002
	Actinomycin	NR peptide	Cytotoxic	Keller et al., 2010
	Alkylresorcinol	Phenolic lipid	–	Funa et al., 2006
	Griseobactin	Catechol-peptide	Siderophore	Patzer and Braun, 2010
ADI98-12	Candicidin	Polyene macrolide	Antifungal	Chen et al., 2003
	Griselimycin	NR peptide	Antibacterial	Kling et al., 2015
	Kirromycin	Polyketide	Antibacterial	Weber et al., 2008

*NR – non-ribosomally synthesized.*

When looking for potential HGT events, we could rather safely suggest those for BGCs specifying RiPPs, ribosomally synthesized and post-translationally modified peptides that may display a wide range of bioactivities (Arnison et al., 2013). Interestingly, we could detect considerable variation in the amino acid sequences of lantipeptide cores, while the thiopeptide primary amino acid sequences were more conserved (Figure 2). This observation may reflect different biological roles that these RiPPs play in their producing hosts, with lantipeptides being more host-specific and thiopeptides more generalistic. Alternatively, thiopeptide BGCs may have been acquired by studied isolates rather recently compared to those for lantipeptides. Notably, the BGCs for PTM were found in 8 out of 10 isolates, and, considering the wide distribution of these clusters in bacteria, their products may play a distinct role in environmental adaptation that is yet to be revealed.

It appeared interesting to compare one of the isolates, ADI95-16, with its terrestrial counterpart *Streptomyces* sp. Mg1, which shared not only homologous chromosomes, but also some linear plasmids. The latter harbor several BGCs, which can be transferred to other species by means of conjugation, thus providing means for distribution of certain BGCs among streptomycetes. Despite the fact that most of the BGCs in the genomes of these strains were identical, several were unique for each strain. It seems likely that the presence of these distinctive BGCs reflects different environments where the strains were isolated from, and signifies requirements for adaptation fulfilled by the production of cognate secondary metabolites.

Our investigation of two bioactive isolates, ADI95-16 and ADI96-02, revealed production of compounds already discovered from terrestrial streptomycetes. However, some new derivatives of linearmycin harboring methylhexose attached to the amino group appear to be produced by ADI95-16. Those have never been reported for the related strain *Streptomyces* sp. Mg1. Since no glycosyltransferase gene could be identified in the linearmycin BGC, it is likely that glycosylation is catalyzed by an enzyme encoded someplace else in the ADI95-16 genome, while the same enzyme is absent or not expressed in Mg1. Notably, the BGC for echinomycin and echinoserine, the compounds produced by ADI96-02, was found to be different in at least three genes compared to echinomycin BGC from *S. lasaliensis*. It is possible, that at least the presence of an additional monooxygenase gene in the ADI96-02 echinomycin BGC may provide opportunities for derivatives with altered oxidation pattern.

Taking together, the results of this study further support the significance of *Streptomyces* bacteria for future drug discovery, and open possibilities for targeted genome mining by means of activation or heterologous expression of selected BGCs. Partial marine adaptation of several isolates suggested by the data presented in Table 1 may also be advantageous in terms of using these strains for heterologous expression of BGCs originating from marine actinomycetes.

## Data Availability Statement

The Whole Genome Shotgun projects have been deposited at DDBJ/ENA/GenBank under the accessions RPGS00000000 (ADI91-18), RPGT00000000 (ADI92-24), RPGU00000000 (ADI93-02), CP033581-CP033585 (ADI95-16), RPGV00000000 (ADI95-17), RPGW00000000 (ADI96-02), RPGX00000000 (ADI96-15), RPGY00000000 (ADI97-07), RPGZ00000000 (ADI98-10), and RPHA00000000 (ADI98-12).

## Author Contributions

SZ conceptualized the study and supervised the project. SZ, MZ, and HB worked on the methodology. CR, TB, and JK were responsible for the software. JG-G, MZ, OS, CR, and HB carried out the investigation. CR, TB, and JK were responsible for the data curation. JG-G, OS, MZ, CR, and SZ prepared and wrote the original draft.

## Conflict of Interest

HB was employed by the company Xellia Pharmaceuticals AS.

The remaining authors declare that the research was conducted in the absence of any commercial or financial relationships that could be construed as a potential conflict of interest.

The handling Editor declared a past co-authorship with one of the authors SZ.

## Footnotes

1 https://www.ezbiocloud.net/

2 https://www.mzcloud.org/DataViewer#Creference2623

3 https://www.mzcloud.org/DataViewer#Creference7689
