# Supplementary material for: Streptomyces spp. From the Marine Sponge Antho dichotoma: Analyses of Secondary Metabolite Biosynthesis Gene Clusters and Some of Their Products
Source: Front Microbiol. 2020 Mar 18;11:437. doi: 10.3389/fmicb.2020.00437 (PMC7093587; doi:10.3389/fmicb.2020.00437)
Supplement: Supplementary file 1 [file Data_Sheet_1.docx]

**Supplemental Material**

***Streptomyces* spp. from the marine sponge *Antho dichotoma*: analyses of secondary metabolite biosynthesis gene clusters and some of their products**

Jaime Felipe Guerrero-Garzón, Martin Zehl, Olha Schneider, Christian Rückert, Tobias Busche, Jörn Kalinowski, Harald Bredholt, Sergey B. Zotchev

**Table S1.** Antibiotic activity of sponge-associated *Streptomyces* spp. grown in 3 different production media.

| **Sample number** | **Strain** | **Medium** | **Day of cultivation** | ***E. coli*** | ***B. subtilis*** | ***Sac. cerevisiae*** |
| --- | --- | --- | --- | --- | --- | --- |
| 1 | 91-18 | PM4 | 3D |  |  |  |
| 2 |  | 5280 |  |  | 1+ | 1+ |
| 3 |  | 5333 |  |  | 1+ | 1+ |
| 4 |  | PM4 | 5D |  |  |  |
| 5 |  | 5280 |  |  | 2+ | 1+ |
| 6 |  | 5333 |  |  |  |  |
| 7 |  | PM4 | 7D |  |  |  |
| 8 |  | 5280 |  |  | 1+ | 1+ |
| 9 |  | 5333 |  |  | 1+ |  |
| 10 | 93-02 | PM4 | 3D |  |  |  |
| 11 |  | 5280 |  |  |  |  |
| 12 |  | 5333 |  |  |  |  |
| 13 |  | PM4 | 5D |  |  |  |
| 14 |  | 5280 |  |  |  |  |
| 15 |  | 5333 |  |  |  |  |
| 16 |  | PM4 | 7D |  |  |  |
| 17 |  | 5280 |  |  |  |  |
| 18 |  | 5333 |  |  |  |  |
| 19 | 95-16 | PM4 | 3D |  | 1+ | 1+(inhibition) |
| 20 |  | 5280 |  |  |  | 1+(inhibition) |
| 21 |  | 5333 |  |  | 1+ | 1+(inhibition) |
| 22 |  | PM4 | 5D |  |  | 1+(inhibition) |
| 23 |  | 5280 |  |  |  |  |
| 24 |  | 5333 |  |  | 1+ | 1+(inhibition) |
| 25 |  | PM4 | 7D |  |  | 1+(inhibition) |
| 26 |  | 5280 |  |  |  |  |
| 27 |  | 5333 |  |  | 1+ | 2+(inhibition) |
| 28 | 95-17 | PM4 | 3D |  |  |  |
| 29 |  | 5280 |  |  |  |  |
| 30 |  | 5333 |  |  |  |  |
| 31 |  | PM4 | 5D |  |  |  |
| 32 |  | 5280 |  |  |  |  |
| 33 |  | 5333 |  |  |  |  |
| 34 |  | PM4 | 7D |  |  |  |
| 35 |  | 5280 |  |  |  |  |
| 36 |  | 5333 |  |  |  |  |
| 37 | 96-02 | PM4 | 3D |  |  | 3+ |
| 38 |  | 5280 |  | 1+(inhibition) | 4+ |  |
| 39 |  | 5333 |  | 1+(inhibition) | 3+ | 4+ |
| 40 |  | PM4 | 5D |  |  | 2+ |
| 41 |  | 5280 |  |  | 4+ |  |
| 42 |  | 5333 |  |  | 3+ | 4+ |
| 43 |  | PM4 | 7D |  |  |  |
| 44 |  | 5280 |  | 1+(inhibition) | 3+ |  |
| 45 |  | 5333 |  | 1+ | 4+ | 3+ |
| 46 | 96-15 | PM4 | 3D |  |  |  |
| 47 |  | 5280 |  |  |  |  |
| 48 |  | 5333 |  |  |  |  |
| 49 |  | PM4 | 5D |  |  |  |
| 50 |  | 5280 |  |  |  |  |
| 51 |  | 5333 |  |  |  |  |
| 52 |  | PM4 | 7D |  |  |  |
| 53 |  | 5280 |  |  |  |  |
| 54 |  | 5333 |  |  |  |  |
| 55 | 97-07 | PM4 | 3D |  |  |  |
| 56 |  | 5280 |  |  |  |  |
| 57 |  | 5333 |  |  |  |  |
| 58 |  | PM4 | 5D |  |  |  |
| 59 |  | 5280 |  |  |  |  |
| 60 |  | 5333 |  |  |  |  |
| 61 |  | PM4 | 7D |  |  |  |
| 62 |  | 5280 |  |  |  |  |
| 63 |  | 5333 |  |  |  |  |
| 64 | 98-10 | PM4 | 3D |  | 2+ |  |
| 65 |  | 5280 |  |  |  |  |
| 66 |  | 5333 |  |  |  |  |
| 67 |  | PM4 | 5D |  | 4+ |  |
| 68 |  | 5280 |  |  | 1+ |  |
| 69 |  | 5333 |  |  | 2+ |  |
| 70 |  | PM4 | 7D |  | 3+ |  |
| 71 |  | 5280 |  |  | 1+ |  |
| 72 |  | 5333 |  |  | 2+ |  |
| 73 | 98-12 | PM4 | 3D |  |  |  |
| 74 |  | 5280 |  |  |  |  |
| 75 |  | 5333 |  |  |  |  |
| 76 |  | PM4 | 5D |  |  |  |
| 77 |  | 5280 |  |  |  |  |
| 78 |  | 5333 |  |  |  |  |
| 79 |  | PM4 | 7D |  |  |  |
| 80 |  | 5280 |  |  |  |  |
| 81 |  | 5333 |  |  |  |  |
| 82 | 92-24 | PM4 | 3D |  |  |  |
| 83 |  | 5280 |  |  |  |  |
| 84 |  | 5333 |  |  |  |  |
| 85 |  | PM4 | 5D |  |  |  |
| 86 |  | 5280 |  |  |  |  |
| 87 |  | 5333 |  |  |  |  |
| 94 |  | PM4 | 7D |  |  |  |
| 95 |  | 5280 |  |  |  |  |
| 96 |  | 5333 |  |  |  |  |

**Table S2. Overview of the assembly and annotation statistics for the 10 sponge-associated *Streptomyces* spp. genomes.**

|  | **ADI91-18** | **ADI92-24** | **ADI93-02** | **ADI95-16** | **ADI95-17** | **ADI96-02** | **ADI96-15** | **ADI97-07** | **ADI98-10** | **ADI98-12** |
| --- | --- | --- | --- | --- | --- | --- | --- | --- | --- | --- |
| **assembly level** | **scaffold** | **scaffold** | **scaffold** | **complete** | **scaffold** | **scaffold** | **scaffold** | **scaffold** | **scaffold** | **scaffold** |
| **coverage** | **63.1x** | **71.5x** | **61.8x** | **158.0x** | **65.4x** | **64.8x** | **61.4x** | **66.3x** | **66.9x** | **68.2x** |
| **sequence length** | **8,639,978** | **9,312,630** | **8,410,972** | **9,086,421** | **10,032,992** | **6,989,279** | **7,085,913** | **8,346,702** | **7,723,553** | **7,243,116** |
| **GC content, %** | **72.3** | **70.6** | **71.1** | **72.2** | **70.6** | **72.2** | **73.2** | **71.0** | **71.4** | **73.4** |
| **# scaffolds** | **8** | **18** | **14** | **5** | **22** | **2** | **10** | **9** | **9** | **10** |
| **scaffold N50** | **2,240,869** | **2,590,990** | **1,497,230** | **n.a.** | **940,612** | **6,907,715** | **1,143,693** | **2,567,091** | **4,397,121** | **1,598,443** |
| **scaffold L50** | **2** | **2** | **2** | **n.a.** | **4** | **1** | **2** | **2** | **1** | **2** |
| **# contigs** | **339** | **295** | **225** | **5** | **363** | **197** | **377** | **201** | **298** | **269** |
| **contig N50** | **53,788** | **67,629** | **77,782** | **n.a.** | **66,096** | **67,230** | **46,611** | **78,044** | **44,943** | **65,000** |
| **contig L50** | **43** | **38** | **36** | **n.a.** | **44** | **32** | **48** | **32** | **56** | **33** |
| **# CDS** | **7,559** | **8,126** | **7,315** | **8,076** | **8,722** | **5,946** | **6,045** | **7,272** | **6,885** | **5,944** |
| **# tRNAs** | **67** | **72** | **69** | **89** | **57** | **57** | **57** | **46** | **65** | **73** |
| **# ncRNAs** | **32** | **15** | **3** | **33** | **22** | **3** | **17** | **4** | **66.9x** | **17** |
| **# BGCs** | **34** | **28** | **29** | **39** | **36** | **30** | **30** | **33** | **28** | **29** |

**Figure S1.** Evolutionary relationship between the 51 Streptomyces isolates from Antho dichotoma inferred using Neighbor-Joining method in MEGA7.0 software..

**Figure S2.** Genome synteny: *Streptomyces* sp. ADI98-12 vs. *S. albus* J1074

**Figure S3.** Genome synteny: *Streptomyces* sp. ADI95-16 vs. *Streptomyces* sp. Mg1

**Figure S4**. Genome synteny: *Streptomyces* sp. ADI96-02 vs. *S. fulvissimus* DSM 40593


**Figure** **S5.** Genome synteny: *Streptomyces* sp. ADI93-02 vs. *Streptomyces* sp. ADI96-02

**Figure S6.** LC-MS data of the bioactive CHCl_3_/MeOH extract of *Streptomyces* spp. ADI95-16: base peak chromatogram (BPC) in the range *m/z* 50-2000 (A), extracted ion chromatogram (EIC) of *m/z* 1140.7193±0.0100 (B), EIC of *m/z* 1166.7350±0.0100 (C), EIC of *m/z* 1316.7878±0.0100 (D), and EIC of *m/z* 1342.8034±0.0100 (E).

**Figure S7.** LC-MS data of the bioactive CHCl_3_/MeOH extract of *Streptomyces* spp. ADI95-16: base peak chromatogram (BPC) in the range *m/z* 50-2000 (A), extracted ion chromatogram (EIC) of *m/z* 1140.7193±0.0100 (B), EIC of *m/z* 1166.7350±0.0100 (C), EIC of *m/z* 1316.7878±0.0100 (D), and EIC of *m/z* 1342.8034±0.0100 (E). The peak shapes suggest the presence of several isomers for each sum formula.

**Figure S8.** LC-MS data of the bioactive CHCl_3_/MeOH extract of *Streptomyces* spp. ADI95-16: measured isotopic pattern of the [M+H]^+^ ion of the tentatively identified linearmycin A (A), calculated isotopic pattern of the [M+H]^+^ ion of linearmycin A with the sum formula C_64_H_101_NO_16_ (B), measured isotopic pattern of the [M+H]^+^ ion of the tentatively identified linearmycin B (C), and measured isotopic patterns of the [M+H]^+^ ions of the new linearmycins tentatively identified as methylhexosylated linearmycin A (D) and methylhexosylated linearmycin B (E).

**Figure S9.** LC-MS data of the bioactive CHCl_3_/MeOH extract of *Streptomyces* spp. ADI95-16: MS/MS spectrum of the [M+H]^+^ ion of the tentatively identified linearmycin A (A), MS/MS spectrum of the [M+H]^+^ ion of the tentatively identified linearmycin B (B), and MS/MS spectrum of the [M+H]^+^ ions of the new linearmycin tentatively identified as methylhexosylated linearmycin A (C).

**Figure S10.** LC-MS data of the bioactive CHCl_3_/MeOH extract of *Streptomyces* spp. ADI95-16: MS/MS spectrum (zoomed y axis) of the [M+H]^+^ ion of the tentatively identified linearmycin A (A), MS/MS spectrum (zoomed y axis) of the [M+H]^+^ ion of the tentatively identified linearmycin B (B), and MS/MS spectrum (zoomed y axis) of the [M+H]^+^ ions of the new linearmycin tentatively identified as methylhexosylated linearmycin A (C).

**Figure S11.** LC-MS data of the bioactive CHCl_3_/MeOH extract of *Streptomyces* spp. ADI96-02: MS spectrum summed over peak **3** (A) and MS/MS spectrum of the [M+H]^+^ ion of the tentatively identified cycloheximide at *m/z* 282.1705 (B).

**Figure S12.** LC-MS data of the bioactive CHCl_3_/MeOH extract of *Streptomyces* spp. ADI96-02: MS spectrum summed over peak **2** (A) and MS/MS spectrum of the [M+H]^+^ ion of the tentatively identified cycloheximide isomer at *m/z* 282.1705 (B).

**Figure S13.** LC-MS data of the bioactive CHCl_3_/MeOH extract of *Streptomyces* spp. ADI96-02: MS spectrum summed over peak **5** (A) and MS/MS spectrum of the [M+H]^+^ ion of the tentatively identified actiphenol at *m/z* 276.1237 (B).

**Figure S14.** LC-MS data of the bioactive XXX extract of *Streptomyces* spp. ADI96-02: MS spectrum summed over peak **1** (A) and MS/MS spectrum of the [M+H]^+^ ion of the tentatively identified nocardamine at *m/z* 601.3594 (B).

**Figure S15.** LC-MS data of the bioactive CHCl_3_/MeOH extract of *Streptomyces* spp. ADI96-02: MS spectrum summed over peak **4** (A) and MS/MS spectrum of the [M+H]^+^ ion of the tentatively identified echinoserine at *m/z* 1137.4522 (B).

**Figure S16.** LC-MS data of the bioactive CHCl_3_/MeOH extract of *Streptomyces* spp. ADI96-02: MS spectrum summed over peak **6** (A) and MS/MS spectrum of the [M+H]^+^ ion of the unambiguously identified echinomycin at *m/z* 1101.4303 (B).

**Figure S17.** LC-MS data of the bioactive CHCl_3_/MeOH extract of *Streptomyces* spp. ADI96-02: BPCs in the range *m/z* 1000-1500 of the original extract (A), the echinomycin reference standard (B), and the ADI96-02 extract spiked with the echinomycin reference standard (C).

**Figure S18.** Proposed fragmentation pathway of the [M+H]^+^ ion of the tentatively identified linearmycin A. The depicted structures for the fragment ions are based on the sum formulae derived from the accurate masses and indicate the most likely position(s) of bond cleavage - however, usually several isomers are possible and no calculations were performed to find the lowest energy structure.

**Figure S19.** Proposed fragmentation pathway of the [M+H]^+^ ion of the tentatively identified linearmycin B. The depicted structures for the fragment ions are based on the sum formulae derived from the accurate masses and indicate the most likely position(s) of bond cleavage - however, usually several isomers are possible and no calculations were performed to find the lowest energy structure.

**Figure S20.** Proposed fragmentation pathway of the of the [M+H]^+^ ion of the new linearmycin tentatively identified as methylhexosylated linearmycin A (part I). The depicted structures for the fragment ions are based on the sum formulae derived from the accurate masses and indicate the most likely position(s) of bond cleavage - however, usually several isomers are possible and no calculations were performed to find the lowest energy structure (R=2xH+1xCH_3_).

**Figure S21.** Proposed fragmentation pathway of the of the [M+H]^+^ ion of the new linearmycin tentatively identified as methylhexosylated linearmycin A (part II). The depicted structures for the fragment ions are based on the sum formulae derived from the accurate masses and indicate the most likely position(s) of bond cleavage - however, usually several isomers are possible and no calculations were performed to find the lowest energy structure (R=2xH+1xCH_3_).

**Figure S22.** Proposed fragmentation pathway of the [M+H]^+^ ion of the tentatively identified cycloheximide. The depicted structures for the fragment ions are based on the sum formulae derived from the accurate masses and indicate the most likely position(s) of bond cleavage - however, usually several isomers are possible and no calculations were performed to find the lowest energy structure.

**Figure S23.** Proposed fragmentation pathway of the [M+H]^+^ ion of the tentatively identified actiphenol. The depicted structures for the fragment ions are based on the sum formulae derived from the accurate masses and indicate the most likely position(s) of bond cleavage - however, usually several isomers are possible and no calculations were performed to find the lowest energy structure.

**Figure S24.** Proposed fragmentation pathway of the [M+H]^+^ ion of the tentatively identified nocardamine. The depicted structures for the fragment ions are based on the sum formulae derived from the accurate masses and indicate the most likely position(s) of bond cleavage - however, usually several isomers are possible and no calculations were performed to find the lowest energy structure.

**Figure S25.** Proposed fragmentation pathway of the [M+H]^+^ ion of the tentatively identified echinoserine. The depicted structures for the fragment ions are based on the sum formulae derived from the accurate masses and indicate the most likely position(s) of bond cleavage - however, usually several isomers are possible and no calculations were performed to find the lowest energy structure.

**Figure S26.** Proposed fragmentation pathway of the [M+H]^+^ ion of the tentatively identified echinomycin. The depicted structures for the fragment ions are based on the sum formulae derived from the accurate masses and indicate the most likely position(s) of bond cleavage - however, usually several isomers are possible and no calculations were performed to find the lowest energy structure.
